# Dataset of shallow sub-surface soil moisture and soil temperature at various distances from downed trees and logs in a *Pinus nigra* forest after wildfire in Central Italy

**DOI:** 10.1016/j.dib.2024.111080

**Published:** 2024-10-28

**Authors:** Flavio Taccaliti, Alessandro Vitali, Carlo Urbinati, Raffaella Marzano, Emanuele Lingua

**Affiliations:** a Università degli Studi di Padova, Dipartimento Territorio e Sistemi Agro-Forestali, Viale dell'Università 16, 35020 Legnaro (PD), Italy; b Università Politecnica delle Marche, Dipartimento di Scienze Agrarie, Alimentari ed Ambientali, Via Brecce Bianche 10, 60131 Ancona, Italy; c Università degli Studi di Torino, Dipartimento di Scienze Agrarie, Forestali e Alimentari, Largo Paolo Braccini 2, 10095 Grugliasco (TO), Italy

**Keywords:** Biological legacies, Coarse woody debris, Log erosion barriers, Pinus nigra J. F. Arnold, Soil moisture, Soil temperature, Wildfire

## Abstract

In a conifer forest in Central Italy burnt by wildfire in 2017, shallow sub-surface (topmost 5 cm) soil temperature and soil moisture (% volumetric water content) were measured during summer 2022. Various distances from downed trees (natural barriers) and log erosion barriers (artificial barriers) were sampled. Additional data on the hour of sampling, barriers characteristics, and barriers location were collected.

Specifications Table

**Table:** 

Subject	Forestry
Specific subject area	Natural disturbances in forest ecosystems
Type of data	Table
Data collection	We took three readings of shallow sub-surface soil moisture and temperature (Delta-T HH2 moisture meter with SM150T probe, Testo 108 thermocouple) near 19 log erosion barriers (artificial b.), and 14 naturally fallen trees (natural b.). We sampled along six sampling positions (five for artificial b.) along transects perpendicular to the barriers. Sampling positions distance was relative to each barrier height.
Data source location	City: Urbino, Marche Country: Italy *Latitude and longitude of collected samples (bounding box): 43.7200° N, 12.7300° E, 43.7100° N, 12.7100° E*
Data accessibility	Repository name: Research Data Unipd Data identification number: 00,001,334 Direct URL to data: https://researchdata.cab.unipd.it/1334/
Related research article	

## 1 Value of the Data

• This dataset presents primary data on shallow sub-surface soil moisture and soil temperature adjacent to log barriers (both natural and artificial) in a coniferous forest after wildfire, data rarely available with this scale of spatial and temporal detail.
• This dataset may be useful to researchers in Forestry and Environmental Sciences investigating the effect of biological legacies or bioengineering techniques on soil in burnt conifer forests.
• Other researchers may use these data in further analyses (e.g., meta-analyses, more refined analyses) examining the effect of biological legacies on the post-fire forest environment.
• This dataset can additionally be used for educational purposes for students in Environmental Sciences or Forestry, and also to test Machine Learning approaches on environmental data, given the size of the dataset.

## 2 Background

**Fig. 1:**
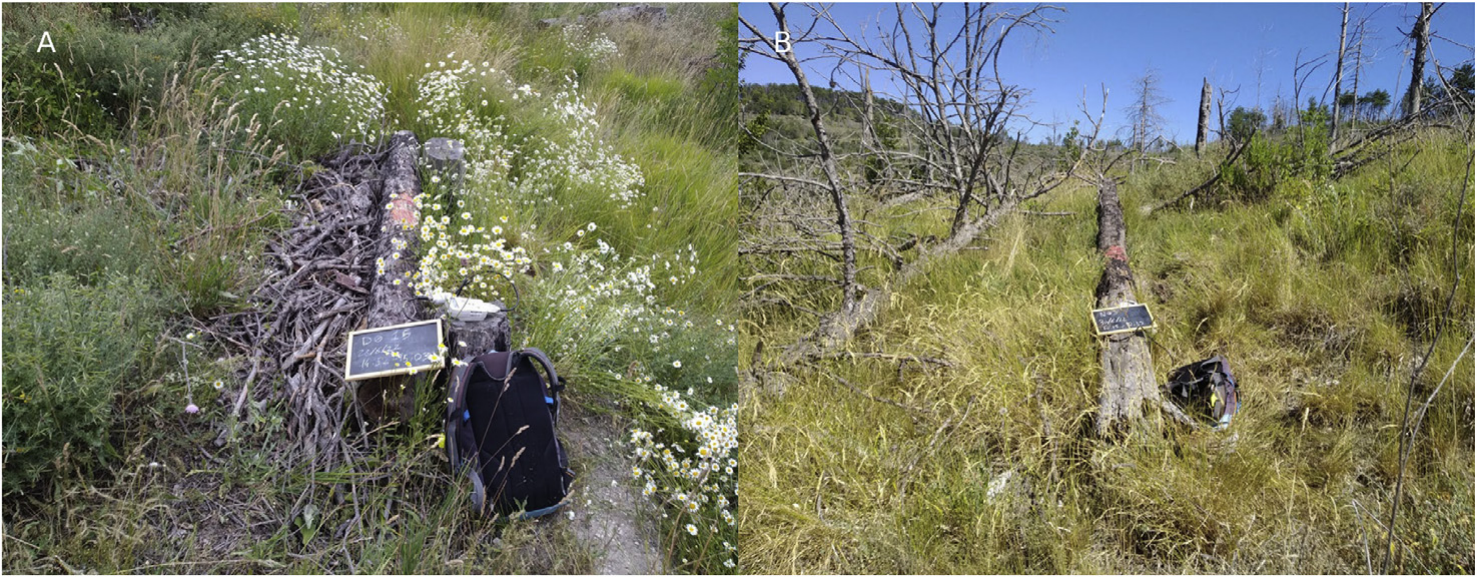
A) artificial log erosion barriers, B) natural barriers originated from fallen trees.

The study site is in the Foresta Demaniale Regionale delle Cesane, Marche, Italy (43.72° N, 12.73° E), a forest mainly composed of *Pinus nigra* J.F. Arnold planted during the 20th century [1]. In July 2017 a wildfire burnt almost 180 ha of forest, of which, 50 ha burned at high severity. The study site is located inside the high-severity area. One year after the fire, the managing authority implemented log erosion barriers (LEBs) to reduce soil erosion: fire-killed pines were cut at 0.5–1 m from the ground, then one, or two, 4–5 m long logs from the trees were placed along the contour lines against the stumps remained after felling. The branches derived from these operations were piled on the uphill side of the LEBs (Fig. 1a). In the years after the fire the remaining trees started to fall due to wind and root wood decay, in some cases creating natural barriers with their boles (Fig. 1b). Raw data of soil parameters near coarse woody debris are scarce, especially in temperate *Pinus nigra* J.F. Arnold forests, while the relation between biological legacies and micro-environment is important in understanding forest regeneration dynamics. Providing raw data allows readers and reviewers to compare different case studies and helps generalise the outcomes of data analysis.

## 3 Data Description

This dataset reports shallow sub-surface soil moisture and soil temperature measured at various distances from natural and artificial log erosion barriers.

Explanation of the variables in the dataset is given below:

• BarrierID. Univocal ID for each of the 33 barriers. ID D06 is missing (removed).
• Type. Type of barrier, either trees naturally fallen after the wildfire (natural, *n* = 14), or log erosion barriers built one year after the wildfire (artificial, *n* = 19).
• Sampling_position. Position relative to the barrier where soil moisture and temperature were sampled.
• Under_log: the closest to the point of contact between the lowest log of the artificial barrier, or the naturally fallen tree, and the ground
• Flush_uphill, _downhill: the points where the vertical projections of the barrier sides meet the ground, on both sides of the barrier
• Downhill, uphill: at a distance from the flush_uphill, flush_downhill positions equal to the barrier height (which corresponds to trees diameter, for natural barriers), on both sides
• Control: in a control point along the barrier longitudinal axis, ca. 2 m from one of its ends, on undisturbed ground
• Moisture_1, _2, _3_VWC. Soil moisture as percent volumetric water content of soil (three measurements).
• Temperature_1, _2, _3_C. Soil temperature in degrees Celsius (three measurements).
• Date. Date of sampling.
• Time_begin, _end. Hour of start and end of sampling at each barrier in each sampling date.
• Barrier_height_cm. Barrier height in centimetres. In case of natural barriers, it corresponds to trees diameter. In case of artificial barrier, to the total height of the barrier.
• Azimuth barrier. Direction of the barrier relative to the magnetic North (declination 3.90° E).
• Terrain slope. Average slope in degrees of the terrain 1 metre around sampling position “under_log”. Calculated from 0.2 m resolution Digital Terrain Model.
• Terrain aspect. Average aspect of the terrain 1 metre around sampling position “under_log”. Calculated from 0.2 m resolution Digital Terrain Model.
• Latitude. Latitude in degrees North of the sampling position “under_log”.
• *Longitude. Longitude in degrees East of the sampling position “under_log”.*

## 4 Experimental Design, Materials and Methods

For both soil moisture, and soil temperature, three readings were taken at each combination of barrier, sampling position, and sampling date. Between readings, the probes were moved parallel to the barrier at a short distance (< 5 cm) from the previous point.

Soil moisture was measured with a Delta-T HH2 moisture meter with a SM150T probe (Delta-T Devices Ltd.). The sensor was calibrated before the first sampling date, and set for measuring mineral soil. When in the field, the whole length of the probe needles was inserted in the ground. In case the probe did not read any moisture (error message), the measurement was taken again, at a short distance (< 5 cm) from the previous point, until three readings were taken.

Soil temperature was measured with a Testo 108 probe (Testo). The probe needle was inserted until its narrowing (about half of the needle) in the ground, to explore the same soil depth of soil moisture. The temperature recorded corresponded to the first stable value on the instrument screen.

At least 36 h were waited after rainfall greater than 10 mm to sample soil moisture and temperature, as in the case of 30 August 2022.

The barriers were identified in two sampling dates, 28 June 2022 (artificial barriers) and 30 June 2022 (natural barriers). On the first day of sampling, the position of each barrier centre (above “under_log” sampling position, UL) was measured with a Topcon HiPer VR GNSS antenna with Real Time Kinematic correction (subcentimetric precision). In the same occasion, barrier ID and transect line (see below) were marked with forestry-grade spray paint on the barrier.

For each barrier, a transect perpendicular to its longitudinal axis was identified, in order to place the sampling positions. Among the barriers available in the study sites, the ones selected to create this dataset had two characteristics: they were laying on the ground for at least one metre on both sides of the transect, and they had no prominent ecological features, such as branches, shrubs, extruded roots, other barriers, etc. for at least 2 m around the intersection of the transect and the barrier longitudinal axis. Also the nearest end of the barrier was at least 2 m from the intersection.

**Fig. 2:**
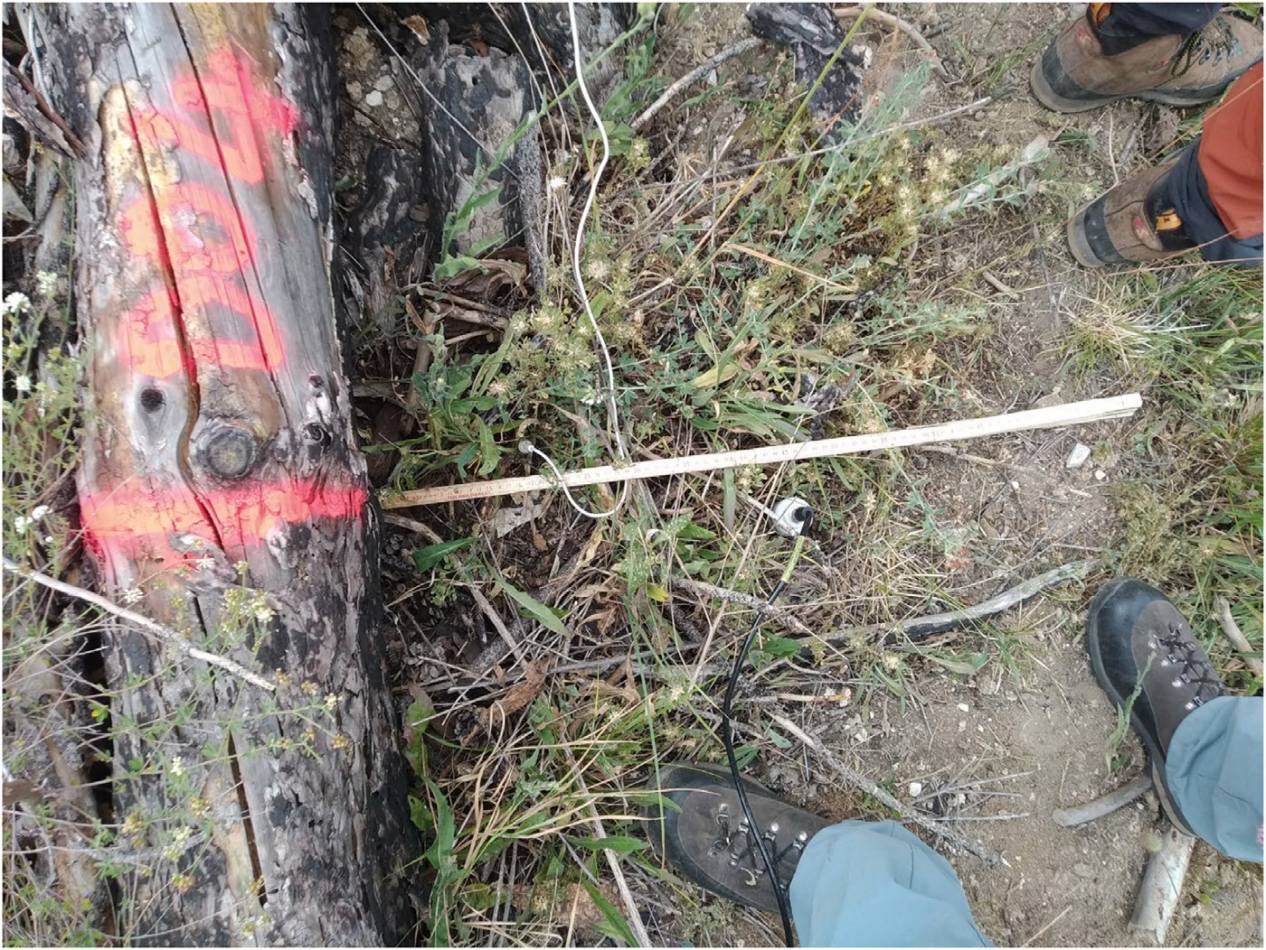
measurement of soil moisture (black cable) and soil temperature (white cable) along the sampling transect. In this case, soil temperature is measured in the position “flush_downhill” (the picture is not perfectly taken from nadir), while soil moisture is measured in the position “downhill”.

Along the transect, the six sampling positions were located as explained in the Data Description section (Fig. 2). For the artificial barriers, no measurements were taken in the sampling position “flush_uphill”, because during the construction of the barriers, branches were accumulated in this position, hindering the access to the soil underneath; for the same reason, the “uphill” position for the artificial barriers was moved at the end of the pile of branches, resulting in a greater distance from the barrier than the downhill position.

Barrier height was measured with a carpenter ruler on the downhill side of the barrier (distance from UL, on the ground, and the top of the barrier). Azimuth was measured with a compass laying on the barrier and aligned with its longitudinal axis.

Slope and aspect of the terrain 1 m around UL was calculated in QGIS with native algorithms from a 0.2 m resolution Digital Terrain Model available for the area.

## Limitations

Not applicable

## Ethics Statement

The authors declare that the current work does not involve human subjects, animal experiments, or any data collected from social media platforms.

## CRediT authorship contribution statement

**Flavio Taccaliti:** Conceptualization, Data curation, Investigation. **Alessandro Vitali:** Supervision, Investigation. **Carlo Urbinati:** Supervision. **Raffaella Marzano:** Conceptualization. **Emanuele Lingua:** Conceptualization, Supervision.

## Data Availability

Research Data UNIPDNear-surface soil temperature and moisture around log erosion barriers and coarse woody debris in a burnt Pinus nigra forest. (Original data)
